# Sir2 and Glycerol Underlie the Pro-Longevity Effect of Quercetin during Yeast Chronological Aging

**DOI:** 10.3390/ijms241512223

**Published:** 2023-07-31

**Authors:** Francesco Abbiati, Stefano Angelo Garagnani, Ivan Orlandi, Marina Vai

**Affiliations:** 1 Dipartimento di Biotecnologie e Bioscienze, Università di Milano-Bicocca, Piazza della Scienza 2, 20126 Milano, Italy; f.abbiati1@campus.unimib.it (F.A.); s.garagnani@campus.unimib.it (S.A.G.); ivan.orlandi@unimib.it (I.O.); 2 SYSBIO Centre for Systems Biology, 20126 Milano, Italy

**Keywords:** quercetin, chronological aging, Sir2, glycerol catabolism, *Saccharomyces cerevisiae*, trehalose

## Abstract

Quercetin (QUER) is a natural polyphenolic compound endowed with beneficial properties for human health, with anti-aging effects. However, although this flavonoid is commercially available as a nutraceutical, target molecules/pathways underlying its pro-longevity potential have yet to be fully clarified. Here, we investigated QUER activity in yeast chronological aging, the established model for simulating the aging of postmitotic quiescent mammalian cells. We found that QUER supplementation at the onset of chronological aging, namely at the diauxic shift, significantly increases chronological lifespan (CLS). Consistent with the antioxidant properties of QUER, this extension takes place in concert with a decrease in oxidative stress. In addition, QUER triggers substantial changes in carbon metabolism. Specifically, it promotes an enhancement of a pro-longevity anabolic metabolism toward gluconeogenesis due to improved catabolism of C2 by-products of yeast fermentation and glycerol. The former is attributable to the Sir2-dependent activity of phosphoenolpyruvate carboxykinase and the latter to the L-glycerol 3-phosphate pathway. Such a combined increased supply of gluconeogenesis leads to an increase in the reserve carbohydrate trehalose, ensuring CLS extension. Moreover, QUER supplementation to chronologically aging cells in water alone amplifies their long-lived phenotype. This is associated with intracellular glycerol catabolism and trehalose increase, further indicating a QUER-specific influence on carbon metabolism that results in CLS extension.

## 1. Introduction

Quercetin (3,3′,4′,5,7-pentahydroxyflavone) (QUER) is a natural polyphenolic compound belonging to flavonols, a sub-class of flavonoids. Its name comes from the Latin word “quercetum”, meaning forest of oaks. However, QUER occurrence, as a secondary metabolite, is widely distributed among plants where it is involved in different physiological processes from seed germination to pollen growth and in providing plant tolerance against some biotic and abiotic stresses [1]. QUER is among the abundant major naturally occurring flavonoids in the human diet via vegetables and fruits. It is found in fruits such as apples, cherries, and berries (blueberries and cranberries) and vegetables such as asparagus, broccoli, peas, green peppers, and onions [2,3,4,5]. In particular, onions (red varieties) have a high content of QUER (about 1.31 mg/100 g of fresh weight) [6].

In 2010, the American Food and Drug Administration notified high-purity QUER as “Generally Recognized as Safe” (GRAS) [7]. Currently, QUER is marketed as a dietary supplement with various claims and statements concerning its health benefits [7]. Indeed, the biological beneficial properties of QUER are well documented, encompassing its direct antioxidant activity to that of modulating signal transduction pathways [8]. In this context, QUER displays, among others, anti-inflammatory, immunoprotective, neuroprotective, anti-carcinogenic and anti-aging effects [2,3,9]. These health-promoting effects are supported by studies performed in eukaryotic model systems, as well as in animals and humans [10,11,12]. The budding yeast *Saccharomyces cerevisiae* is a single-celled eukaryote that, exploited as a model system, has proved to help decipher conserved fundamental processes/pathways of multicellular eukaryotes, despite the large evolutionary distance involved. As far as human aging/longevity is concerned, in this yeast, two complementary aging models allow us to simulate the cellular aging of actively proliferating cells, exemplified by fibroblasts, and that of post-mitotic, albeit metabolically active cells, namely myocytes [13,14,15]. The former is the replicative aging model, the latter the chronological aging one. Chronological lifespan (CLS) is the length of time (mean and maximum) that a culture of non-dividing cells remains viable in the stationary phase: viability is estimated by the ability to resume growth and form a colony upon return to a rich, fresh medium [16]. In a standard CLS experiment, yeast cells are grown in synthetic media with 2% glucose [16]. In this condition, growth predominantly relies on a fermentation-based metabolism that allows a fast depletion of available glucose and concomitantly provides an extracellular accumulation of metabolites, particularly the extensive release of ethanol in the medium. Only when glucose becomes limiting does the diauxic shift occur, and cells undergo a highly regulated transition from a fermentative to a respiratory metabolism in which by-products of the fermentation become substrates and are consumed. The diauxic shift is the turning point between two distinct metabolic states; it is characterized by structural, functional, and physiological rearrangements that involve a huge rewiring of gene expression resulting, among others, in an increase in the metabolic flux through the TCA cycle, in the onset of the glyoxylate shunt and of gluconeogenesis. The last one switches carbon flux toward reserve carbohydrates [17,18]. This contributes, along with antioxidant defence systems, to establish a proper protective state of quiescence that ensures the long-term survival of non-dividing cells during the stationary phase and resumes growth upon refeeding. The ability of cells to perform such rearrangements through a series of interlocking signaling networks is a fundamental aspect that strongly affects CLS [19,20,21]. TORC1-Sch9 and Ras-PKA pathways are two nutrient-sensing pathways that negatively regulate the transition into quiescence, and their inhibition/inactivation at different levels extends CLS [18,22].

On the contrary, impairing the activity of the glyoxylate/gluconeogenic pathway and the accumulation of reserve carbohydrates (in particular trehalose) reduces CLS [20,23,24]. In this context, the NAD^+^-dependent deacetylase Sir2, which is the founding member of Sirtuins, deacetylates phosphoenolpyruvate carboxykinase (Pck1) [24], the activity of which is the main flux-controlling step of gluconeogenesis. Pck1 is active in the acetylated form [24], and *SIR2* deletion correlates with an increase in the acetylated active Pck1, enhancing gluconeogenesis and trehalose content in concert with CLS extension [23,25]. In chronologically aging *sir2*∆ cells, all this is accompanied by a decrease in oxidative stress [26]. The same outcomes are detected after nicotinamide (NAM) supplementation at the diauxic shift [26]. NAM, which is a form of vitamin B_3_, is an endogenous non-competitive inhibitor of the deacetylation reaction catalyzed by Sirtuins, including Sir2; it shifts the enzymatic reaction toward the reformation of NAD^+^ and acetylated target(s) [27,28]. Concerning NAM-supplemented cells, an increase in the acetylated Pck1 is observed due to the lack of Sir2-targeted deacetylation [26]. Conversely, opposite outcomes are detected after resveratrol (RSV) supplementation at the diauxic shift [29]. RSV, a natural non-flavonoid polyphenolic compound, restricts CLS and increases oxidative stress. In RSV-supplemented cells, a reduction of the acetylated Pck1 is observed in concert with a decrease in gluconeogenesis and trehalose stores [29].

Here, we focused on the effects of QUER supplementation at the diauxic shift. The results indicate that QUER determines CLS extension accompanied by a decrease in oxidative stress in line with its inbuilt characteristics of antioxidants. In addition, we show that QUER deeply influences carbon metabolism allowing cells to acquire features useful for better survival during chronological aging. In particular, QUER improves the assimilation of the C2 by-product of yeast fermentation through Sir2-dependent Pck1 activity and glycerol catabolism resulting in trehalose increase. Furthermore, QUER also extends CLS under extreme Calorie Restriction (CR, chronologically aging cells in water) together with enhancement of intracellular glycerol catabolism and trehalose stores, indicating that critical components of the beneficial effects of QUER on CLS are changes in carbon metabolism.

## 2. Results and Discussion

### 2.1. Quercetin Supplementation at the Diauxic Shift Extends CLS and Promotes Trehalose Accumulation

**Figure 1:**
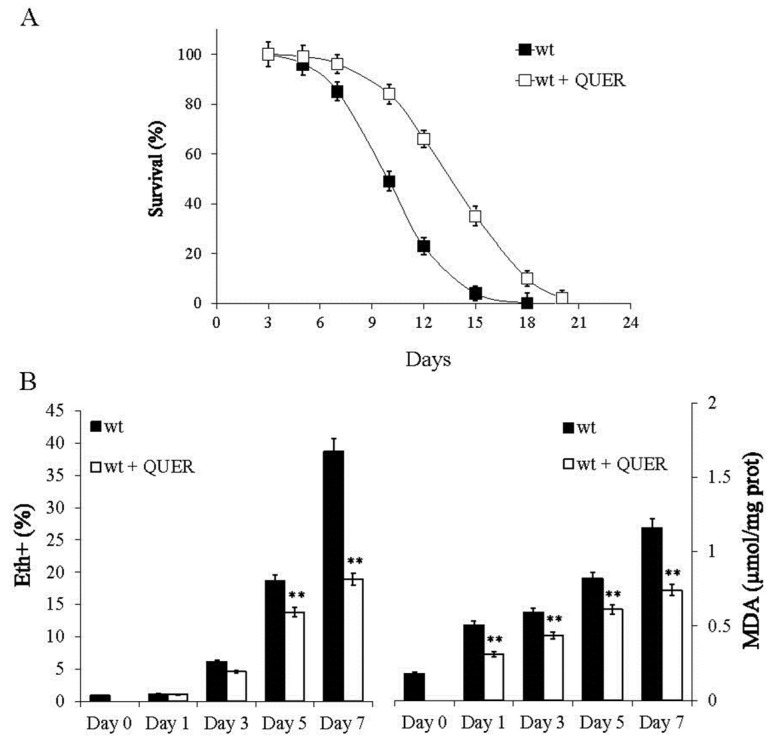
QUER supplementation at the diauxic shift extends CLS and reduces oxidative stress. Wild-type (wt) cells were grown in minimal medium/2% glucose and the required supplements in excess (see Materials and Methods). At the diauxic shift (Day 0), quercetin (QUER, 300 µM) was added, and (**A**) survival over time of treated and untreated cultures was assessed by colony-forming capacity on YEPD plates. 72 h after the diauxic shift (Day 3) was considered the first age point, corresponding to 100% survival. In parallel, for the same cultures (**B**) left: bar charts of the percentage of fluorescent/superoxide positive cells assessed by the superoxide-driven conversion of non-fluorescent dihydroethidium into fluorescent ethidium (Eth) and right: intracellular malondialdehyde (MDA) concentration. All data refer to mean values determined in three independent experiments with three technical replicates each. Standard deviations (SD) are indicated. Statistical significance as assessed by a one-way ANOVA test is indicated (** *p* ≤ 0.01).

Since previous works reported that QUER treatment to yeast cells exponentially growing on glucose increases CLS [30,31,32,33], we set out to evaluate whether a similar positive effect could be observed following its supplementation at the onset of chronological aging, namely at the diauxic shift. At the diauxic shift, cells shift from glucose-driven fermentation to ethanol/acetate-driven respiration, and the outcomes of such a metabolic reconfiguration influence CLS [19,20,21,34,35]. QUER-supplemented cells displayed an increase of both mean and maximum CLS (Figure 1A and Table S1) as well as of the survival integral (Table S1) defined as the area under the CLS curves and calculated according to [36], compared to unsupplemented cells.

The CLS extension was accompanied by decreased levels of two oxidative stress biomarkers, such as superoxide anion (O_2_^·−^) and malondialdehyde (MDA) (Figure 1B). The former is one of the most potentially harmful ROS, and the latter is a natural end-product of lipid peroxidation: both accumulate as chronological aging progresses, limiting cellular longevity [35,37,38]. The antioxidant activity is a feature shared with other flavonoids and is linked to the chemical structure of this class of molecules, which allows them to scavenge free radicals directly. In addition, the antioxidant property of flavonoids relies on their ability to chelate metal ions, mainly iron ones [39,40]. Iron plays a crucial role in many metabolic processes, and Fe^2+^ participates in the generation of free radicals in the Fenton reaction contributing to oxidative stress. However, in yeast, a decrease in oxidative stress markers (ROS, glutathione oxidation, protein carbonylation and lipid peroxidation) observed after QUER treatment was not associated with iron chelation suggesting that this beneficial effect of QUER is independent of its intrinsic iron-chelating properties [33].

**Figure 2:**
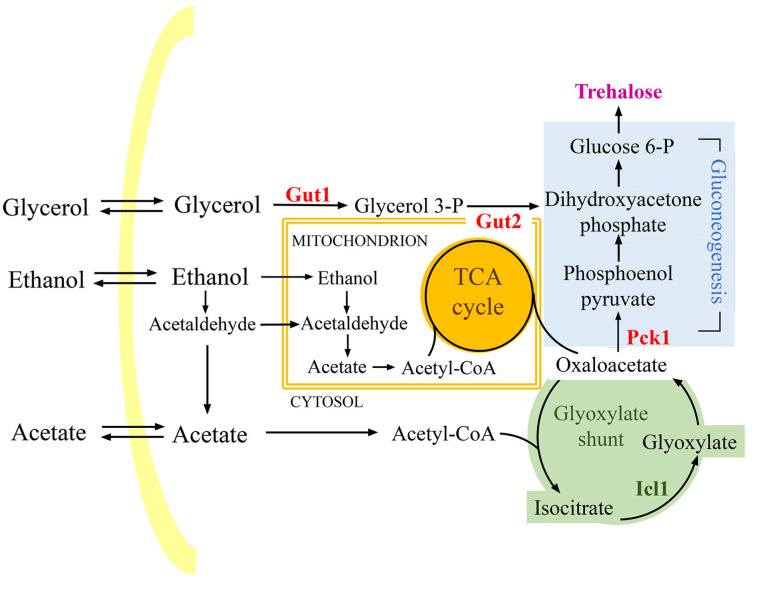
Scheme of metabolic pathways involved in utilising the main non-fermentable carbon sources during chronological aging. Three pathways (TCA cycle, glyoxylate shunt and gluconeogenesis) are schematically shown. Gut1, glycerol kinase; Gut2, mitochondrial glycerol-3-phosphate dehydrogenase; Pck1, phosphoenolpyruvate carboxykinase; Icl1, isocitrate lyase.

**Figure 3:**
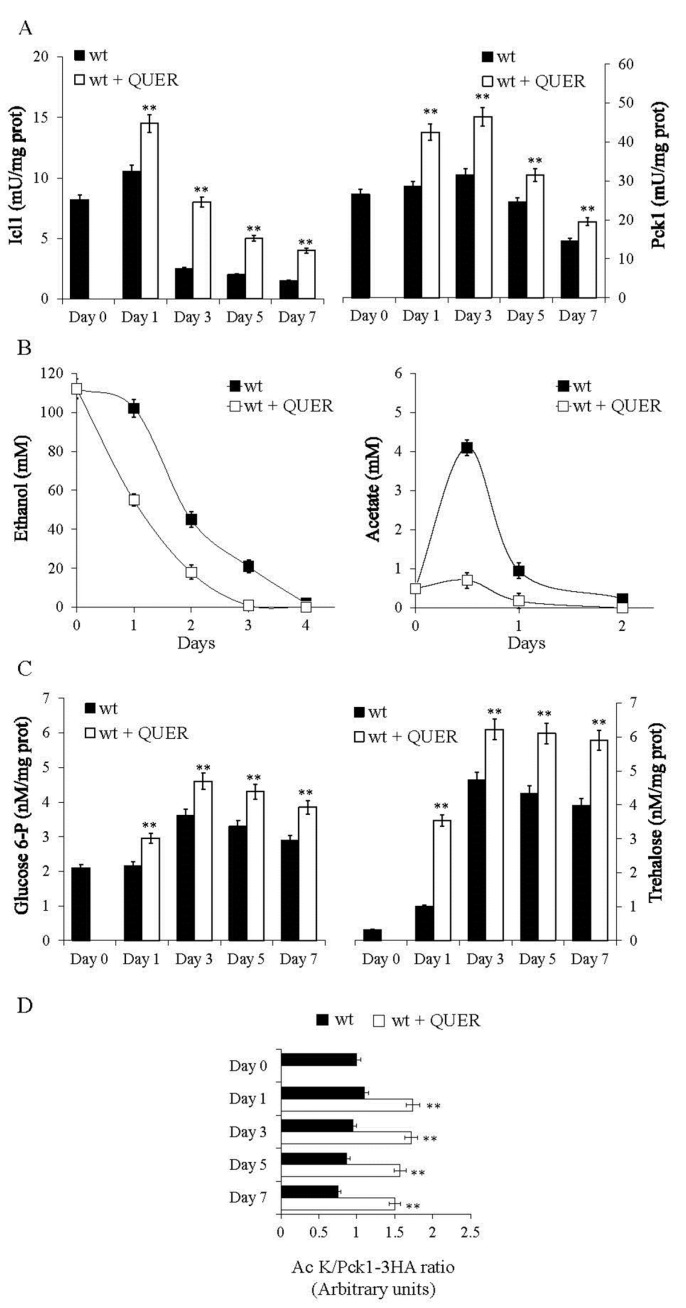
QUER supplementation at the diauxic shift enhances the glyoxylate/gluconeogenic flux. At the indicated time points, (**A**) Icl1 and Pck1 enzymatic activities, (**B**) ethanol and acetate levels and (**C**) glucose-6-phosphate (G6P) content along with trehalose one evaluated for both treated and untreated cultures of Figure 1. (**D**) Bar charts of the ratio of Ac-K (acetylated form of Pck1) to Pck1-3HA (total Pck1) values obtained by densitometric quantification of signal intensity of the corresponding bands on Western blots. Wt cells expressing Pck1-3HA were grown and supplied with QUER, as in Figure 1. At different time points, total protein extracts were prepared from treated and untreated cultures and subjected to immunoprecipitation with anti-HA antibodies, followed by Western analysis. Immunodecoration was performed with anti-Ac-K and anti-HA antibodies. All data refer to mean values determined in three independent experiments with three technical replicates each. SD is indicated (** *p* ≤ 0.01).

Starting from the aforementioned results, which align with QUER’s antioxidant and anti-aging properties, we decided to analyze the metabolic changes underlying the beneficial effects of QUER supplementation at the diauxic shift. Initially, we measured the enzymatic activity of isocitrate lyase (Icl1), one of the unique enzymes of the glyoxylate shunt, and that of Pck1, the key enzyme of gluconeogenesis. The glyoxylate shunt and the gluconeogenesis are anabolic pathways operative during chronological aging and involve using ethanol and acetate (Figure 2). These are C2 compounds, the metabolism of which influences CLS [14,41,42]. In QUER-supplemented cells, the enzymatic activities of Icl1 and Pck1 were higher than those in the unsupplemented ones (Figure 3A), consistent with increased utilization of ethanol and acetate (Figure 3B), indicating an enhancement of glyoxylate/gluconeogenic pathways. Gluconeogenesis allows the production of glucose-6-phosphate (G6P), which is required for trehalose biosynthesis. In QUER-supplemented cells, increased G6P levels and trehalose ones were detected (Figure 3C). Trehalose is a disaccharide stored by chronologically aging cells and is advantageous for their survival [20,43]. In keeping with this finding, gene expression profiles of QUER-treated cells in the exponential phase showed upregulation of genes involved in trehalose biosynthesis associated with an increase in trehalose content and acquisition of oxidative stress resistance [33].

Moreover, as far as Pck1 activity is concerned, its increase in QUER-supplemented cells was associated with an increase in the level of the acetylated active form of the enzyme (Figure 3D). Since Pck1 activity is inhibited by Sir2-mediated deacetylation [24,25,26,29], we wondered whether QUER could affect Sir2 activity. To this end, an α-factor sensitivity assay was performed.

**Figure 4:**
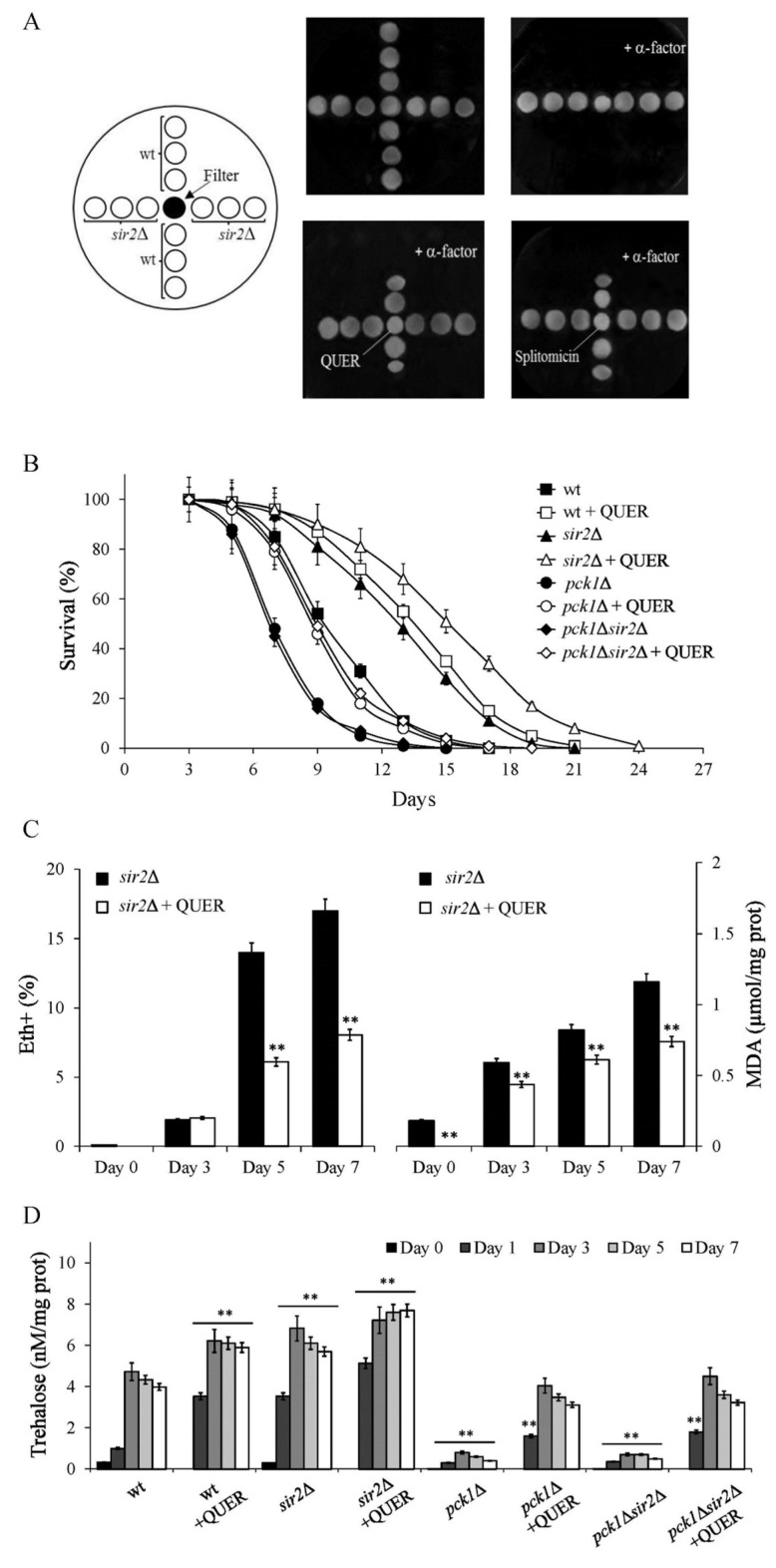
QUER inhibits Sir2 activity. (**A**) Wt and *sir2*Δ exponentially growing cells were dropped (5 µL of a 10^6^ cells/mL dilution) onto glucose-rich/medium plates (top left), supplemented with 2.5 µM α-factor (top right and bottom left and right). A concentration gradient of QUER was formed by loading 5 µL of 300 µM QUER on a filter disk placed on the agar (bottom left). 5 µL of 5 mM splitomicin was loaded on a filter disk as a control (bottom right). Growth was monitored after three days at 30 °C. (**B**) CLS of the indicated strains grown and supplied with QUER as in Figure 1. (**C**) Bar charts of the percentage of intracellular superoxide accumulating cells (Eth) and intracellular malondialdehyde (MDA) concentration. Strains are indicated. (**D**) Intracellular trehalose concentration was evaluated for the indicated cultures. All data refer to mean values determined in three independent experiments with three technical replicates each. SD is shown (** *p* ≤ 0.01).

Sir2 deacetylase activity is essential for gene silencing at *HM loci,* and in a haploid strain, its absence determines a pseudodiploid state [44]. Thus, in the presence of α-factor, *MATa* wild type (wt) cells were arrested in the G1 phase of the cell cycle and did not grow, whilst *sir2*∆ cells grew as they were unresponsive to the pheromone (Figure 4A). Notably, in the presence of QUER, wt cells lost sensitivity to α-factor and no longer grew in the presence of the pheromone. No effect was observed on *sir2*∆ cells (Figure 4A). Similar behaviour was observed for wt cells in the presence of splitomicin, used as a control (Figure 4A). This compound inhibits Sir2 deacetylase activity and creates a conditional phenocopy of a *sir2*∆ mutant [45]. All these data suggest that in the presence of QUER, Sir2 is inhibited, and a pro-longevity anabolic metabolism toward gluconeogenesis and trehalose storage takes place.

To further assess if Sir2 activity is involved in QUER-mediated outcomes, we analyzed the consequences of QUER supplementation at the diauxic shift in the absence of the deacetylase. As previously reported, *SIR2* deletion extended CLS (Figure 4B, Table S1) [23,26,29,46,47] in concert with a reduction of oxidative stress biomarkers (Figure 4C) and increased trehalose levels (Figure 4D) [23,26,29]. Interestingly, QUER supplementation amplified the CLS extension of the *sir2*Δ mutant (Figure 4B, Table S1), as well as the decrease of oxidative stress biomarkers (Figure 4C) and the increase of trehalose (Figure 4D). Thus, due to this synergistic effect of QUER supplementation and *SIR2* deletion, it is reasonable to think that Sir2 may only mediate a portion of the effects of QUER and that this compound may also exert its activity on additional targets.

As stated, trehalose production relies upon gluconeogenesis, controlled by Pck1 enzymatic activity. In addition, gluconeogenesis plays a positive role in CLS extension [24,48]. Indeed, the loss of Pck1 strongly decreased both CLS (Figure 4B, Table S1) [23,24] and trehalose content (Figure 4D). Moreover, *SIR2* deletion did not affect either CLS of *pck1*Δ cells (Figure 4B, Table S1) [23,24] or trehalose levels (Figure 4D), indicating that Pck1 is required for the CLS extension and the increase of trehalose stores of chronologically aging *sir2*Δ cells. On the contrary, supplementing QUER to *pck1*Δ cells or *pck1*Δ*sir2*Δ ones resulted in a CLS identical to that of chronologically aging wt cells, although less than that measured for QUER-supplemented wt cells (Figure 4B, Table S1). Notably, the same effect was observed for trehalose content (Figure 4D), indicating that QUER supplementation at the diauxic shift can also promote gluconeogenesis to some extent, regardless of Pck1. Since this enzyme catalyzes the rate-limiting step in gluconeogenesis by converting oxaloacetate to phosphoenolpyruvate (Figure 2), this implies that in the *pck1*Δ mutant, QUER can also fuel gluconeogenesis with other substrates available during the post-diauxic phase that allow to bypass the requirement of oxaloacetate.

### 2.2. Quercetin Supplementation at the Diauxic Shift Also Enhances Glycerol Catabolism

Glucose-fermenting cells of *S. cerevisiae* produce, in addition to C2 by-products (ethanol and acetate), some glycerol as a by-product for cytosolic redox balancing [49]. Glycerol is transiently accumulated in the culture medium and, after the diauxic shift, is catabolised by the L-glycerol 3-phosphate (L-G3P) pathway [50]. This pathway involves the sequential action of two enzymes: a glycerol kinase encoded by *GUT1* and a L-glycerol-3-phosphate dehydrogenase encoded by *GUT2*. The final product is dihydroxyacetone phosphate that can be channelled into gluconeogenesis, bypassing the step catalyzed by Pck1 (Figure 2). Consequently, in light of the above results, we focused on glycerol catabolism.

**Figure 5:**
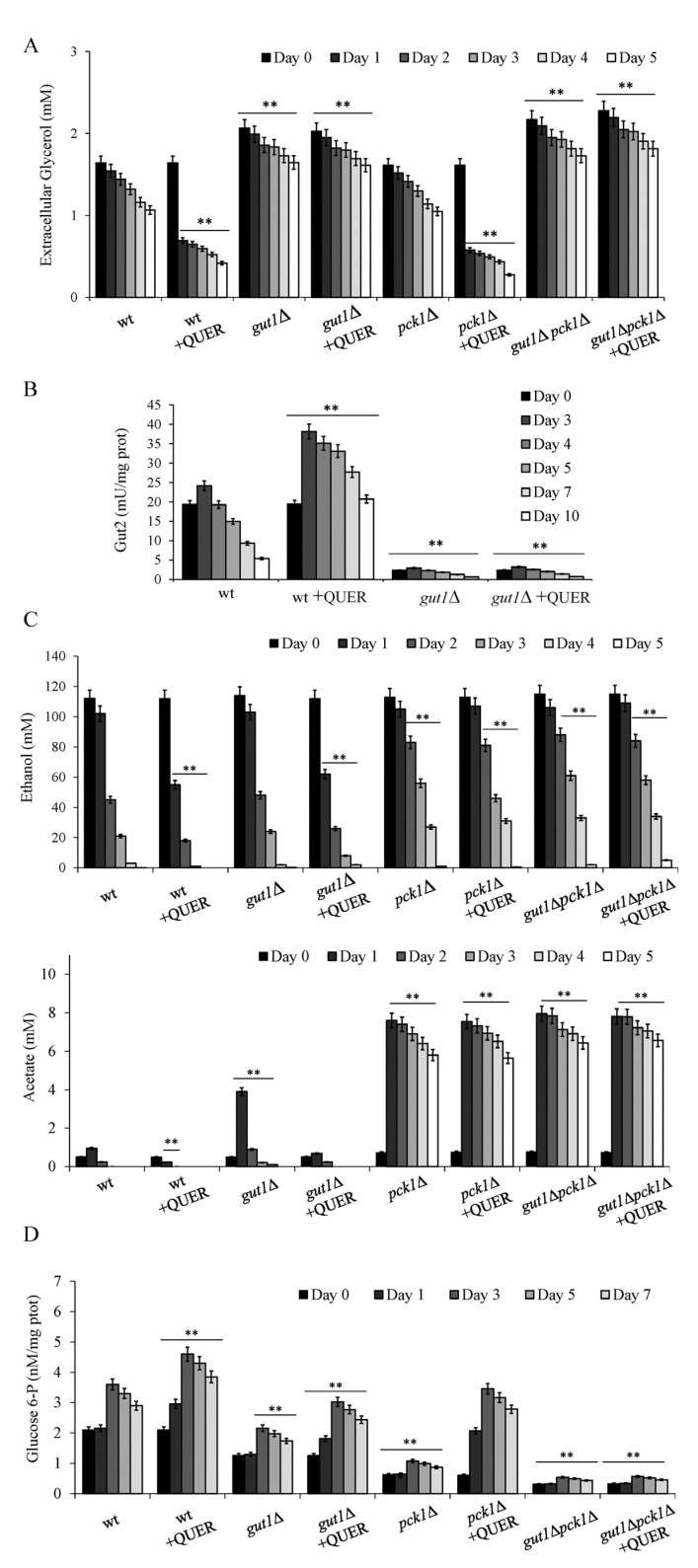
QUER supplementation at the diauxic shift enhances glycerol catabolism. Wt, *pck1*Δ, *gut1*Δ and *gut1*Δ*pck1*Δ cells were grown and supplied with QUER as in Figure 1. At the indicated time points: (**A**) extracellular glycerol levels, (**B**) Gut2 enzymatic activity, (**C**) extracellular ethanol and acetate concentrations and (**D**) glucose-6-phosphate (G6P) content were determined. All data refer to mean values determined in three independent experiments with three technical replicates each. SD is indicated (** *p* ≤ 0.01).

In QUER-supplemented chronologically aging wt cells, intracellular (Figure S1) and extracellular glycerol (Figure 5A) were depleted more rapidly than unsupplemented ones. The same behaviour was observed for chronologically aging *pck1*∆ cells. On the contrary, QUER supplementation to the *gut1*∆ culture had no impact on the levels of both intracellular (Figure S1) and extracellular glycerol (Figure 5A). This was also true for the double deletion mutant (*gut1*∆*pck1*∆) (Figure 5A). These data are in line with previous reports showing that Pck1 is dispensable when *S.cerevisiae* is growing on a C3 substrate such as glycerol [51,52] and suggest that QUER supplementation at the diauxic shift enhances glycerol catabolism. Indeed, the glycerol depletion observed for wt cells in the presence of QUER (Figure 5A) was accompanied by a strong increase in the glycerol-3-phosphate dehydrogenase activity (Figure 5B). In *gut1*∆ cells, this enzymatic activity was almost negligible (Figure 5B) due to the lack of the glycerol kinase converting glycerol to glycerol-3-phosphate, which is the substrate of the dehydrogenase (Figure 2).

Concerning ethanol and acetate, since *GUT1* is not required for growth on these C2 compounds [51], in the *gut1*∆ culture, their kinetics of depletion in the medium were like those of the wt culture, and QUER supplementation led to a similar increase (Figure 5C,D). In line with Pck1 role in the utilization of C2 carbon sources, in chronologically aging *pck1*∆ cells and *gut1*∆*pck1*∆ ones, the depletion of both ethanol and acetate strongly slowed down compared to that of the wt. Furthermore, QUER supplementation had no effect (Figure 5C,D) and this is consistent with the involvement of Pck1 in the increased utilization of ethanol and acetate detected in QUER-supplemented wt cells. In parallel, comparisons of intracellular G6P levels measured in chronologically aging wt cells and in the three mutants clearly showed on the one hand, the different contributions of the L-G3P pathway and the step catalyzed by Pck1 to gluconeogenesis and, on the other, that both routes are required to fuel the gluconeogenic pathway to provide G6P during the post-diauxic phase (Figure 2 and Figure 5D). In this context, very low G6P levels were detected in the *gut1*∆*pck1*∆ mutant and were unaffected by QUER supplementation (Figure 5D). On the contrary, supplementing QUER to *pck1*Δ cells, as well as to *gut1*Δ ones, increased the G6P levels, albeit to a lesser degree than those measured for QUER-supplemented wt cells (Figure 5D), correlating well with the notion that QUER impacts on gluconeogenesis at two entry points.

**Figure 6:**
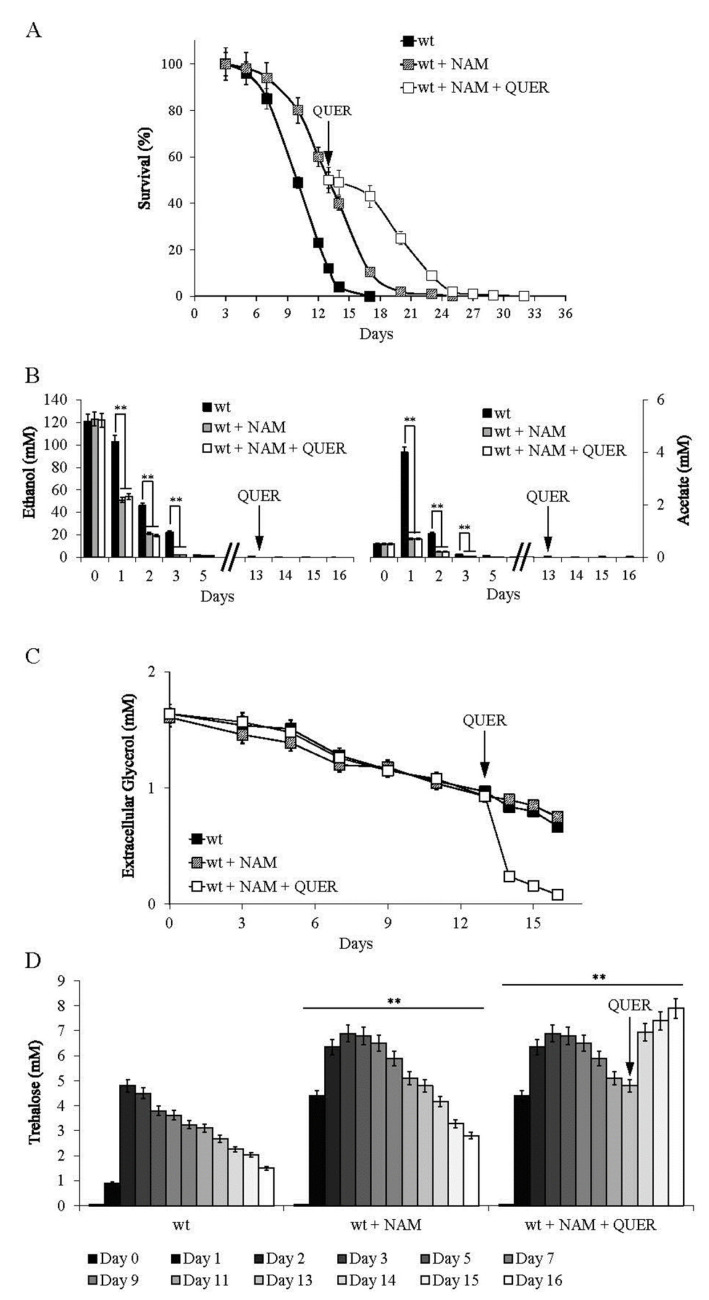
QUER supplementation during chronological aging further extends the CLS of NAM-treated cells. Wt cells were grown as in Figure 1 and supplied with NAM (5 mM) at the diauxic shift (Day 0) At the time-point where NAM stationary cultures showed 50% of survival (mean CLS), QUER (300 µM) was added. (**A**) The CLS of the indicated cultures is determined in Figure 1. In parallel, (**B**) extracellular ethanol and acetate content, (**C**) extracellular glycerol levels and (**D**) intracellular trehalose concentration were measured. All data refer to mean values determined in three independent experiments with three technical replicates each. SD is indicated (** *p* ≤ 0.01).

We found that NAM, a well-known non-competitive inhibitor of Sir2 activity [27], supplemented at the diauxic shift phenocopies chronologically aging *sir2*∆ cells by inhibiting Sir2-mediated deacetylation of Pck1 [26]. This resulted in an increased CLS (Figure 6A and Table S1) and, among others, increased ethanol/acetate catabolism (Figure 6B) [26]. Thus, NAM was supplied to wt cells at the diauxic shift, and when the NAM stationary culture showed 50% of survival (mean CLS), QUER was added (Figure 6A). In the expired medium, ethanol and acetate were exhausted (Figure 6B), whilst glycerol was still present (Figure 6C). Following QUER supplementation a strong decrease in extracellular glycerol was observed compared to NAM-supplemented cells (Figure 6C), further experimentally reinforcing the finding that QUER enhances glycerol catabolism. Concomitantly, trehalose levels increased (Figure 6D), and CLS was extended (Figure 6A), further supporting the positive correlation between these two parameters.

These data indicate that QUER supplementation at the diauxic shift stimulates the gluconeogenic flux through the L-G3P pathway and Pck1 activity. This leads to improved assimilation of C2 by-products of yeast fermentation and glycerol during the post-diauxic phase and increased reserve carbohydrate trehalose, ensuring long-term survival during chronological aging.

### 2.3. Quercetin Enhances Intracellular Glycerol Catabolism, and Further Extends CLS under Extreme CR

**Figure 7:**
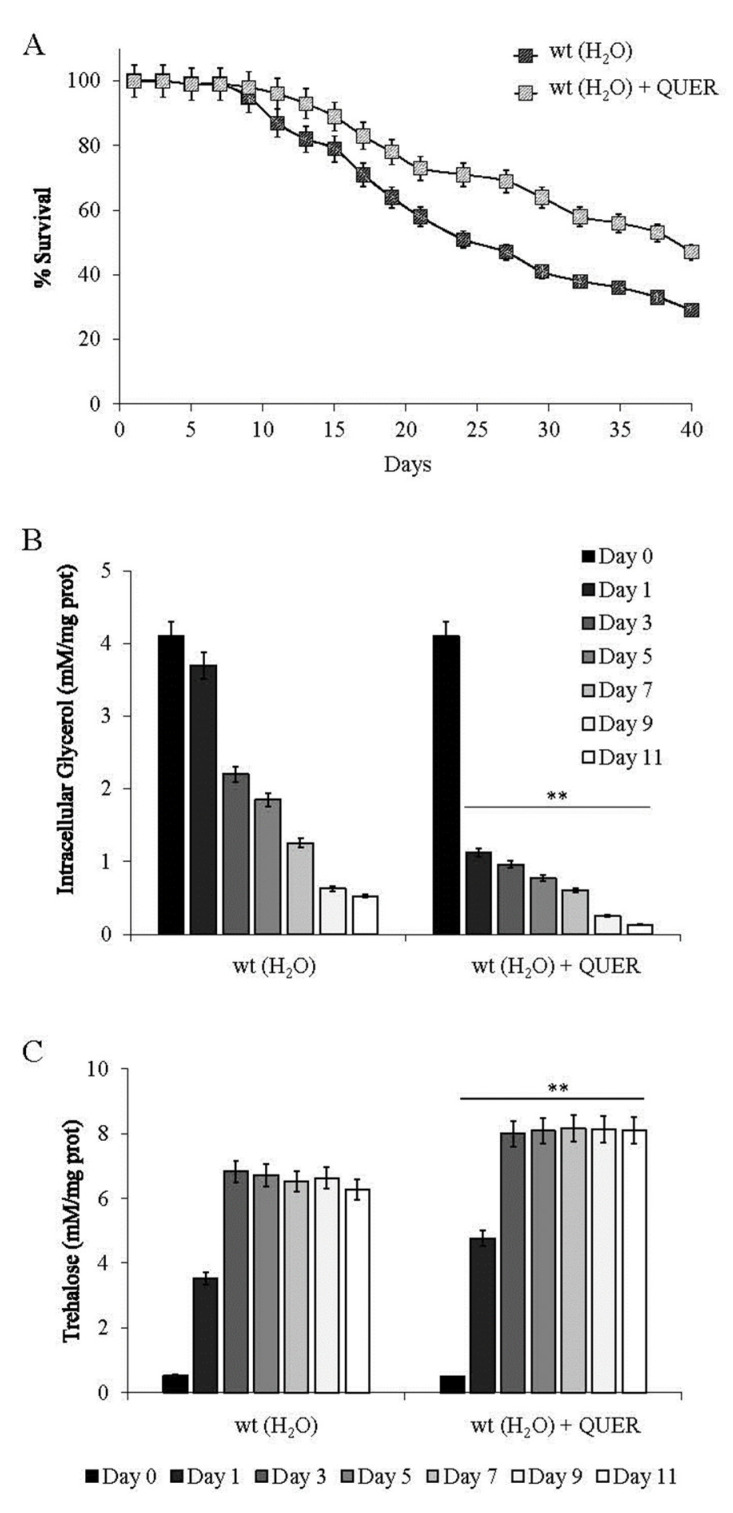
QUER supplementation further enhances CLS extension under extreme CR. At the diauxic shift (Day 0) wt cells (grown as in Figure 1) were switched to water (pH adjusted to 3.2) and challenged with QUER (300 µM). Every 48 h cultures were resuspended in fresh water, and each time QUER was added, they were reported. At the indicated time points (**A**) CLS, (**B**) intracellular glycerol and (**C**) trehalose content of cell cultures were determined in parallel after Day 0. All data refer to mean values determined in three independent experiments with three technical replicates each. SD is indicated (** *p* ≤ 0.01).

Switching post-diauxic yeast cells from expired medium to water models an extreme condition of CR known to extend CLS remarkably [14,42,47] (Figure 7A and Table S1).

In this context, we evaluated the effects of QUER supplementation on wt cells that, after the diauxic shift, were transferred to water, namely in the absence of any extracellular nutrient/carbon/energy source. Supplementing QUER to water amplified the long-lived phenotype of chronological aging cells in water alone (Figure 7A and Table S1). In parallel, measurements of intracellular glycerol and trehalose showed that in the presence of QUER, the utilization of the former increased considerably over time in concert with the increased content of the latter (Figure 7B,C). Hence, QUER brings about trehalose accumulation at the expense of the main compatible solute/osmolyte glycerol, albeit CR-restricted chronological aging cells face an extreme survival-based metabolism. Both trehalose and glycerol are compatible solutes, the accumulation and interplay of which have been shown to occur in yeast during various stress conditions to maximize the probability of cell survival and/or proliferation [53,54,55]. However, trehalose has more specific roles in protecting proteins and preserving membrane structures, along with being the carbohydrate of choice for surviving starvation and upon cell cycle reentry from starvation [20,43]. In line with this, its increase is sufficient to improve an “extreme” CLS further.

Finally, since glycerol can be utilized by *S.cerevisiae* as a sole carbon/energy source under aerobic conditions, we also analyzed QUER effects on the growth behavior of wt cells during exponential growth on this C3 compound. A significant decrease in the Td was detected for cells growing on glycerol in the presence of QUER compared to that measured in its absence (Table 1). Similarly, growth on a non-fermentable substrate such as ethanol occurred faster in the presence of QUER, whilst no effect was observed for cells grown on a fermentable substrate such as glucose (Table 1). Taken as a whole, this further indicates that QUER positively affects the catabolism of glycerol and ethanol. This effect is independent of the physiological state of the cells since it takes place in both actively growing cells and chronological aging ones. In these last cells, an enhancement of the anabolic metabolism toward gluconeogenesis and trehalose storage extends the CLS.

## 3. Materials and Methods

### 3.1. Yeast Strains, Growth Conditions and CLS Determination

All yeast strains used in this work were generated by PCR-based methods in a W303-1A background (*MATa* *ade2-1 his3-11,15 leu2-3,112 trp1-1 ura3-1 can1-100*) and are listed in Table S2. The accuracy of gene replacements and correct deletions/integrations was verified by PCR with flanking and internal primers. Cells were grown in batches at 30 °C in a minimal medium (Difco Yeast Nitrogen Base without amino acids, 6.7 g/L) with the indicated carbon source at 2% (3% for glycerol) and supplements added in excess [56]. Cell number was determined during growth using a Coulter Counter-Particle Count and Size Analyzer [57]. Duplication time (Td) was obtained by linear regression of the cell number increase over time on a semi-logarithmic plot. Survival experiments were performed on cells grown in a minimal medium with 2% *w*/*v* glucose and supplements added in excess. Samples were collected at different time points to define the growth profile (exponential phase, diauxic shift (Day 0), post-diauxic phase and stationary phase) of the culture [56]. CLS was measured according to [47] by counting colony-forming units (CFU) starting with 72 h (Day 3, first age-point) after Day 0. The number of CFU on Day 3 was considered the initial survival (100%). QUER (dissolved in DMSO, purchased from Sigma-Aldrich) was added at the final concentration of 300 µM. NAM (Sigma-Aldrich, Darmstadt, Germany) at the final concentration of 5 mM. Survival experiments in water (pH adjusted to 3.2) were performed as described [41]. Every 48 h, 300 µM of QUER was added to the culture after washing. Viability was determined by CFU.

### 3.2. Dosage of Metabolites and Enzymatic Assays

At designated time points, aliquots of the yeast cultures were centrifuged, and both pellets (washed twice) and supernatants were collected and frozen at −80 °C until used. Rapid sampling for intracellular metabolite measurements was performed as described [56]. The glucose, G6P, ethanol, acetic acid and glycerol concentrations were determined using enzymatic assays (K-HKGLU, K-ETOH, K-ACET and K-GCROL kits from Megazyme, Bray, Ireland). Extraction and determination of intracellular trehalose according to [58]. The K-HKGLU kit was used to quantify the released glucose.

Pck1 and Icl1 activities were assayed as previously reported [56]. FAD-dependent glycerol-3-phosphate dehydrogenase (Gut2) activity was determined according to [59]. Total protein concentration was estimated using the BCA^TM^ Protein Assay Kit (Thermo Fisher Scientific, Waltham, MA, USA).

### 3.3. Estimation of Superoxide Levels and Lipid Peroxidation

Dihydroethidium (DHE, Sigma-Aldrich) staining was performed to analyze superoxide anion (O_2_ˉ) [60]. Cells were counterstained with propidium iodide to discriminate between live and dead cells. A Nikon Eclipse E600 fluorescence microscope equipped with a Nikon Digital Sight DS Qi1 camera was used. Digital images were acquired and processed using Nikon software NIS-Elements (https://www.microscope.healthcare.nikon.com/products/software/nis-elements).

Lipid peroxidation was determined by quantifying MDA using the BIOXYTECH^®^ LPO-586™ Colorimetric Assay Kit (OxisResearch, Portland, OR, USA). The assay is based on the reaction of the chromogenic N-methyl-2-phenylindole with MDA forming a stable chromophore with maximum absorbance at 586 nm.

### 3.4. Immunoprecipitation and Western Analysis

Total protein extract preparation, immunoprecipitation and Western analysis were performed as described [23]. Primary antibodies used were anti-HA (12CA5, Roche, Mannheim, Germany) and anti-acetylated-lysine (Ac-K-103, Cell Signaling, Leiden, The Netherlands). Secondary antibodies were purchased from Amersham. Binding was visualized with the ECL Western Blotting Detection Reagent (Amersham Pharmacia Biotech, Milan, Italy). After ECL detection, films were scanned on a Bio-Rad GS-800 calibrated imaging densitometer and quantified with Scion Image software 4.0.3.2.

### 3.5. HM Silencing Assay

Silencing was examined using an α-factor sensitivity halo assay [61] with some modifications [62]. In brief, drops (5 μL of a 10^6^ cell/mL dilution) of exponentially growing cells were pin-spotted onto agar plates (2% glucose/minimal medium and appropriate supplements) containing α-factor (2.5 μM final concentration). Then, 5 μL of 5 mM splitomicin (dissolved in DMSO) was loaded on a sterile filter disk placed in the centre of the agar plates to form a concentration gradient of splitomicin. Similarly, a concentration gradient of QUER was formed by loading 5 μL of 300 μM QUER. Plates were incubated at 30 °C for 2/3 days. Cells were also dropped onto plates without pheromones and scored for growth. Both α-factor and splitomicin were purchased from Sigma-Aldrich.

### 3.6. Statistical Analysis of Data

All values are presented as the mean of three independent experiments ± Standard Deviation (SD). Three technical replicates were analyzed in each independent experiment. Statistical significance was assessed by a one-way ANOVA test. The level of statistical significance was set at a *p* value of ≤0.05.

## 4. Conclusions

The flavonol QUER is endowed with high antioxidant properties, as proven by many in vivo and in vitro studies, that provide numerous health-promoting benefits, including anti-aging ones. We found that QUER supplementation at the onset of chronological aging, namely at the diauxic shift, extends yeast CLS. This beneficial effect relies on the influence on carbon metabolism induced by QUER, which leads to improved assimilation of C2 by-products of yeast fermentation and glycerol during the post-diauxic phase. It follows an enhancement of a pro-longevity anabolic metabolism toward gluconeogenesis fuelled at two entry points: the L-G3P pathway and Sir2-dependent Pck1 activity. The outcome is increased reserve carbohydrate trehalose, which ensures long-term survival during chronological aging, thus benefiting cellular longevity.

## Figures and Tables

**Table 1:** QUER promotes respiratory metabolism.

Medium	Td (h) *
Glycerol	5.44 ± 0.15
Glycerol + QUER	3.35 ± 0.13
Ethanol	4.30 ± 0.15
Ethanol + QUER	3.00 ± 0.16
Glucose	1.40 ± 0.11
Glucose + QUER	1.40 ± 0.13

Duplication time (Td) of wt culture growing on different carbon sources. * Td was calculated as ln2/k, where k is the constant rate of exponential growth. Data represent the average of three independent experiments. Standard deviations are indicated.

## Data Availability

Not applicable.

## Supplementary Materials

The following supporting information can be downloaded at: https://www.mdpi.com/article/10.3390/ijms241512223/s1.
