# Supplementary material for: Sir2 and Glycerol Underlie the Pro-Longevity Effect of Quercetin during Yeast Chronological Aging
Source: Int J Mol Sci. 2023 Jul 31;24(15):12223. doi: 10.3390/ijms241512223 (PMC10419316; doi:10.3390/ijms241512223)
Supplement: Supplementary file 1 [file ijms-24-12223-s001.zip › ijms-2518818-supplementary.pdf]

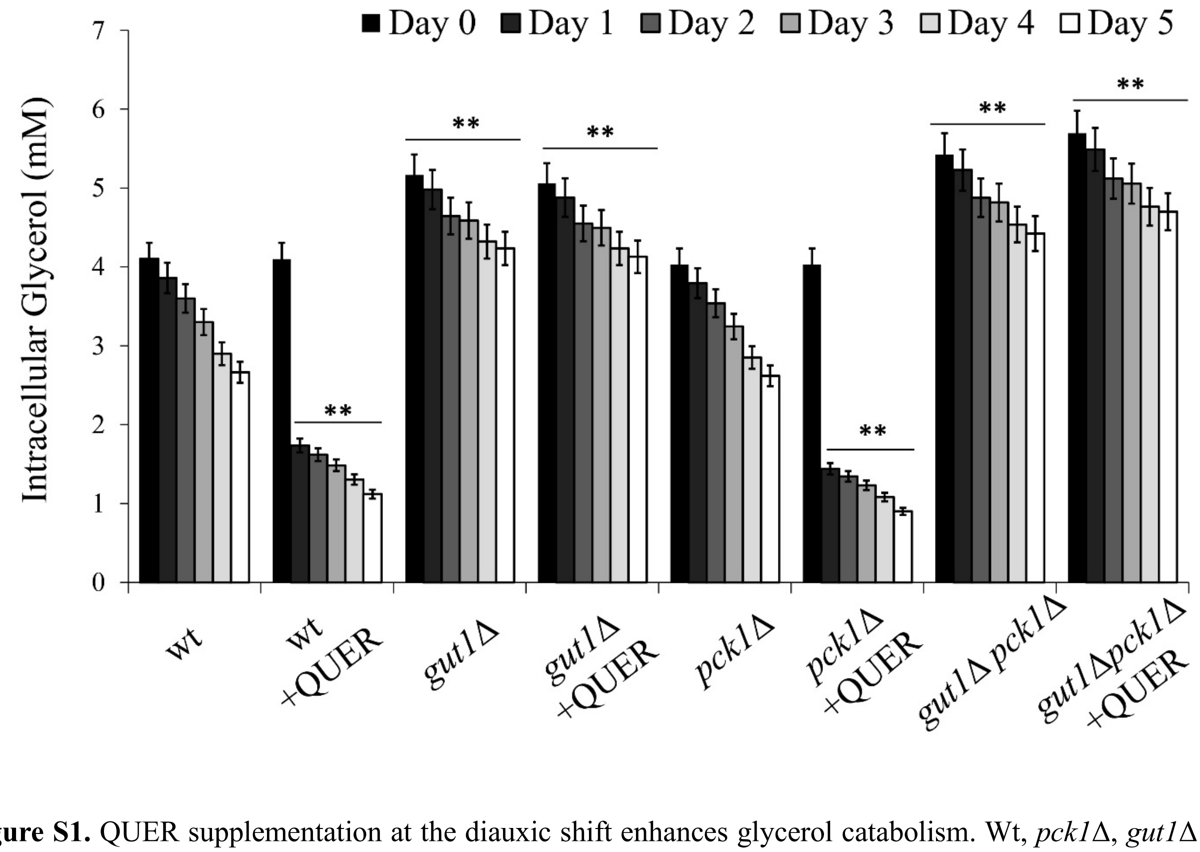

Figure S1. QUER supplementation at the diauxic shift enhances glycerol catabolism. Wt, *pck1*Δ, *gut1*Δ and *gut1*Δ*pck1*Δ cells were grown and supplied with quercetin (QUER, 300 μM) at the diauxic shift. At the indicated time-points intracellular glycerol levels were determined. All data refer to mean values determined in three independent experiments with three technical replicates each. SD is indicated (**P ≤ 0.01)

Table S1: Quantification of chronological survival**.**

Strain	Mean CLS	Max CLS	SI CLS curve
wt (W303-1A)	10.30 ± 0.67	14.70 ± 0.29	685.7 ± 67
wt + QUER	13.55 ± 0.51**	18.60 ± 0.45**	996.07 ± 54**
*sir2*Δ	13.12 ± 0.46**	17.80 ± 0.38**	989.43 ± 75**
*sir2*Δ + QUER	15.19 ± 0.32**	20.79 ± 0.57**	1313.00 ± 43**
*pck1*Δ	6.45 ± 0.53**	9.85 ± 0.49**	436.32 ± 29**
*pck1*Δ + QUER	10.19 ± 0.24	14.20 ± 0.17	676.12 ± 63
*pck1*Δ*sir2*Δ	6.71 ± 0.42**	10.00 ± 0.32**	440.31 ± 39**
*pck1*Δ*sir2*Δ + QUER	9.59 ± 0.35	13.50 ± 0.40	675.32 ± 56
wt + NAM	13.06 ± 0.58**	17.24 ± 0.49**	979.53 ± 34**
wt (H_2O)	23.18 ± 0.61**	41.74 ± 0.61**	1416.76 ± 44**
wt (H_2O) + QUER	27.94 ± 0.84**	58.74 ± 0.83**	2068.41 ± 67**

Data referring to the time points where chronological aging cultures showed 50% (Mean CLS) and 10% (Max CLS) of survival as well as survival integral (SI) measured as reported by [36].

36. Murakami, C.; Kaeberlein, M. Quantifying yeast chronological life span by outgrowth of aged cells. *J. Vis. Exp.* **2009**, *27*, e1156, doi:10.3791/1156.

Table S2: Yeast strains used in this study.

Strain	Relevant genotype	Source
W303-1A	*MAT*a *ade2-1 his3-11,15 leu2-3,112 trp1-1 ura3-1 can1-100*	P.P. Slominski
YVU21	W303-1A *sir2Δ::URA3*	[61]
YVU83	W303-1A *pck1Δ::KlLEU2*	[23]
YVU84	W303-1A *sir2Δ::URA3 pck1Δ::KlLEU2*	[23]
YVU90	W303-1A *PCK1-3HA::KlURA3*	[23]
YVU91	W303-1A *sir2Δ::HIS3 PCK1-3HA::KlURA3*	[23]
YVU97	W303-1A *gut1Δ::HIS3*	This study
YVU98	W303-1A *gut1Δ::HIS3 sir2Δ::URA3*	This study
YVU99	W303-1A *gut1Δ::HIS3 pck1Δ::KlLEU2*	This study

23. Casatta, N.; Porro, A.; Orlandi, I.; Brambilla, L.; Vai, M. Lack of Sir2 increases acetate consumption and decreases extracellular pro-aging factors. *Biochim. Biophys. Acta* **2013**, *1833*, 593–601, doi:10.1016/j.bbamcr.2012.11.008.
60. Calzari, L.; Orlandi, I.; Alberghina, L.; Vai, M. The histone deubiquitinating enzyme Ubp10 is involved in rDNA locus control in *Saccharomyces cerevisiae* by affecting Sir2p association. *Genetics* **2006**, *174*, 2249–2254, doi:10.1534/genetics.106.063099.
